# Identification of Blackberry (*Rubus fruticosus*) Volatiles as *Drosophila suzukii* Attractants

**DOI:** 10.3390/insects12050417

**Published:** 2021-05-06

**Authors:** Peter Dewitte, Vincent Van Kerckvoorde, Tim Beliën, Dany Bylemans, Tom Wenseleers

**Affiliations:** 1 Laboratory of Socioecology and Social Evolution, Department of Biology, KU Leuven, Naamsestraat 59, B-3000 Leuven, Belgium; tom.wenseleers@kuleuven.be; 2 Zoology Department, Research Centre for Fruit Cultivation (pcfruit npo), Fruittuinweg 1, B-3800 Sint-Truiden, Belgium; vincent.vankerckvoorde@pcfruit.be (V.V.K.); tim.belien@pcfruit.be (T.B.); dany.bylemans@pcfruit.be (D.B.); 3 Department of Biosystems, KU Leuven, Decroylaan 42, B-3001 Heverlee, Belgium

**Keywords:** spotted wing drosophila, fruit volatiles, monitoring, mass trapping, olfactory preference, pest management, headspace SPME GC-MS

## Abstract

**Simple Summary:**

The spotted wing drosophila, *Drosophila suzukii*, is an invasive pest species from Southeast Asia that was recently introduced in parts of Europe and North America. As *D. suzukii* lays its eggs in ripening soft-skinned fruit, it causes significant damage to a wide variety of summer fruit, including cherries, blueberries, blackberries, raspberries, grapes, plums and strawberries. Therefore, there is a need for an effective attractant to improve monitoring or allow for mass trapping of this fly. Because blackberry is one of the preferred host crops of *D. suzukii*, the volatiles which this berry emits were analyzed via GC-MS in order to identify the key compounds with an attractive effect. In total, 33 volatiles were tested of which six proved to be significantly attractive to *D. suzukii.* Of these compounds, acetaldehyde, hexyl acetate, linalool and myrtenol proved to be most attractive. Overall, these results can form a valuable basis to further develop more effective and selective lures to monitor or mass trap this pest.

**Abstract:**

The spotted wing drosophila, *Drosophila suzukii*, is an invasive pest species from Southeast Asia that was recently introduced in Europe and North America. As this fruit fly lays its eggs in ripening soft-skinned fruit, it causes great damage to a variety of crops, including cherries, blueberries, blackberries, raspberries, grapes, plums and strawberries. Consequently, there is a great demand for an effective and species-specific lure, which requires the development of successful attractants. Until now, there is no lure available that is species-specific and can detect the presence of *D. suzukii* before infestation. As blackberry (*Rubus fruticosus*) is one of the preferred host crops of *D. suzukii*, the volatile compounds of *R. fruticosus* berries are here identified and quantified using multiple headspace SPME (solid phase micro extraction) GC-MS (gas chromatography–mass spectrometry). Subsequently, the attractivity of 33 of the identified compounds was tested with a two-choice laboratory bioassay. Acetaldehyde, hexyl acetate, linalool, myrtenol, *L*-limonene and camphene came out as significantly attractive to *D. suzukii*. The first four attractive compounds induced the strongest effect and therefore provided the best prospects to be implemented in a potential lure. These findings could contribute towards the development of more effective attractants for monitoring and mass trapping *D. suzukii*.

## 1. Introduction

*Drosophila suzukii* is an emerging pest species that originated in Southeast Asia, but subsequently invaded large parts of Europe and North America, where it is now causing extensive harm to the fruit growing industry. In Europe, *D. suzukii* was first recorded in Spain and Italy in 2008 and 2009 [1,2], after which it quickly spread throughout the rest of western Europe, where it now poses a major threat to the fruit growing industry [3]. In contrast to endemic *Drosophila* species, *D. suzukii* has a serrated ovipositor, which allows it to penetrate the skin of healthy, ripening fruit to lay its eggs. This causes damage due to larval feeding on fruit flesh and increases the susceptibility of the fruit to fungal and bacterial pathogens. The polyphagous nature and broad host range of *D. suzukii* further mean that it causes extensive damage to a wide variety of soft and thin-skinned fruits, including cherries, blueberries, blackberries, raspberries, grapes, plums and strawberries [2,4]. 

The general way by which this pest is managed is via the routine use of broad-spectrum pesticides, which is costly, carries environmental risks, e.g., via carry-over effects on other beneficial insects, and disrupts the integrated pest management of other pests [5]. Other management options, such as exclusion netting [6], the use of repellents [7,8,9,10], sanitation [11,12] or mass trapping [10,13,14,15,16,17,18], have therefore been considered as more environmentally friendly alternatives. Unfortunately, up till now none of these has proven to be sufficiently effective in practice. Essential for any effective integrated pest management program as well as to limit pesticide use is the availability of reliable monitoring traps. Although insect traps are often based on aggregation or sex pheromones, up till now only short-range contact pheromones have been identified in *Drosophila* [18,19,20]. Hence, available fruit fly traps mostly make use of attractive fruit fermentation products, such as wine or apple cider vinegar [18,21,22]. Such traditional attractants lack two important aspects of a good lure. Firstly, they generally fail to timely detect the pest species before the infestation develops, which means the lure needs to be highly attractive when population density is low. Secondly, they often display poor selectivity, implying that such lures also attract many non-target species [14,23]. Recently, significant effort has been put into trying to improve traditional fruit fermentation attractants by adding compounds that improve both their attractiveness as well as their selectivity for *D. suzukii* [18,21]. Some of the commercial lures now available can detect the presence of *D. suzukii* up till 21 days before fruit infestation. However, these only work with specific fruit crops and still have a relatively low selectivity [24]. 

Research has shown that *D. suzukii* uses host plant volatiles as a cue to find food and oviposition sites [25]. Blackberries, blueberries, raspberries and wine grapes are all very susceptible to egg laying by *D. suzukii* [26,27], and among these, blackberries *(Rubus fruticosus)* have been shown to be a preferred host, being the berry which experienced the highest infestation rates [28,29]. To enable the development of more effective and selective lures for monitoring or mass trapping *D. suzukii*, the volatiles emitted by *R. fruticosus* were here identified and quantified using MHS-SPME (multiple headspace Solid Phase Micro Extraction) GC-MS, and the attractivity of the identified compounds to *D. suzukii* was studied. Although SPME is typically not used as a quantitative extraction technique, multiple consecutive extractions on the same sample (MHS-SPME GC-MS) have recently been shown to be an effective means to semi-quantitatively estimate the total amounts of different volatiles released by fruits, vegetables or other food [30,31,32]. All identified and commercially available compounds were subsequently tested for their attractivity to gravid *D. suzukii* females using two-choice laboratory bioassays.

## 2. Materials and Methods

### 2.1. Fly Rearing

The *D. suzukii* population used in the experiments was collected in a rural area in a garden with *Prunus avium* cherries (Breendonk, Belgium, 51.050425° N, 4.333443° E) in May 2019. The flies were subsequently reared in the Laboratory of Socioecology and Social Evolution (KU Leuven, Belgium) in 175 mL containers (Greiner Bio-one, which are containers for plant tissue culture) on a medium consisting of 6% sugar, 1.5% yeast, 0.7% agar, 10% polenta and 0.1% methyl 4-hydroxybenzoate (Sigma-Aldrich, Darmstadt, Germany, CAS 99-76-3) [19], which served as a food source and as a site for oviposition. The flies were reared under a light-dark regime of 16:8 and a constant temperature of 23 ± 1 °C.

### 2.2. Chemical Analysis of R. fruticosus Volatiles

The berries of *R. fruticosus* cv. Lochness originated from a greenhouse at the Research Centre for Fruit Cultivation, pcfruit npo, Sint-Truiden, Belgium (50.462425° N, 5.93779° E). The ripe berries were picked when they were completely black. Afterwards, the berries were cut to fit the 20 ml headspace vials and frozen (−18 °C) until analysis. There were four biological replicates, with each sample containing one berry. To identify and quantify the volatile compounds emitted by *R. fruticosus*, the berry samples were analyzed using MHS- SPME followed by GC-MS (MHS-SPME GC-MS) as in [30,31,32]. The sample was kept at 60 °C under agitation for 5 min using a TriPlus RSH Autosampler (Thermo Fisher Scientific, Waltham, MA, USA). The temperature is increased compared to natural conditions to improve the recovery of the compounds, which is essential for the quantification, while keeping the temperature low enough to avoid artifacts [33]. Following [34,35], the volatiles were extracted by binding to a 50/30 µm DVB/CAR/PDMS coating fiber (Supelco, Darmstadt, Germany) and injected using splitless mode with an inlet temperature of 270 °C, a split flow of 9 mL/min, purge flow of 5mL/min and a splitless time of 3 min. The flow of helium carrier gas was programmed to start at 2.7 mL/min for 0.1 min after which it slowed down with 20 mL/min^2^ until a flow of 0.9 ml/min was reached. The volatiles were separated using a Thermo Trace 1300 GC system (Thermo Fisher Scientific) with an MXT-5 column (30 m length × 0.25 mm inner diameter × 0.25 µm film thickness; Restek, Bellefonte, PA, USA) and an ISQ mass spectrometer (Thermo Fisher Scientific). The oven temperature changed at the following rate: it started at 30 °C for the first 3 min, then it increased with 7 °C/min until the temperature reached 80 °C, afterwards, the temperature increased with 2 °C/min until 125 °C, after which the temperature increased with a rate of 8 °C/min till it reached 270 °C. Mass spectra were recorded in a range of 33-550 amu, using a scan time of 0.2 sec. A mix of linear alkanes (C7-C40 Saturated Alkanes Standard, Supelco, Sigma) were run under the same conditions and served as external calibration to calculate cubic spline interpolated retention indices [36]. From each sample, volatiles were extracted and analyzed over fifteen consecutive runs, by which point most volatiles present in the blackberry sample were exhausted. This MHS-SPME GC-MS method (MHS-SPME GC-MS) allowed us to quantify the total amount of each volatile present in one berry [30,31,32]. 

GC-MS data of the blackberry samples were analyzed as described by Reher et al. [7]. This resulted in 38 unique volatile compounds being identified. In short, CDF files of the chromatograms were analyzed using AMDIS (version 2.71) to deconvolute overlapping peaks. With NIST MS Search (version 2.2), spectra were annotated based on measured retention time, retention index and mass spectral matches to the NIST2011, FFNSC and Adams GC-MS mass spectral libraries. For every individual compound in each sample, the elution profiles were then extracted using weighted non-negative least square analysis [37]. This resulted in calculated peak areas for each compound per sample for each of the fifteen runs. Compounds with peak areas lower than 1000 or peak areas from berry samples that were not significantly higher than in the blank samples were left out. In total, 33 of the 38 identified compounds were commercially available at the time of the experiments. The compounds could be subdivided into five functional groups: thirteen terpenes, eleven alcohols, five aldehydes, two esters and two ketones. 

To estimate the quantity of each compound in one *R. fruticosus* berry, a blend of the 33 available identified compounds, made up in a concentration of 0.01 µL/ml per compound in chloroform (CAS: 67-66-3, VWR, Radnor, PA, USA) solvent, of which 1 µL was injected in liquid mode using a splitless injection at 270 °C (and all other settings as above), was used to calibrate the measured MHS-SPME signal and measure response factors [30]. This external calibration allowed us to convert the total peak areas (total area under the curve) from the blackberry samples over the fifteen subsequent extractions and runs to total absolute amounts. All the identified compounds with corresponding supplier can be found in the supplementary materials (Table S1). 

### 2.3. Experimental Set-Up

**Figure 1:**
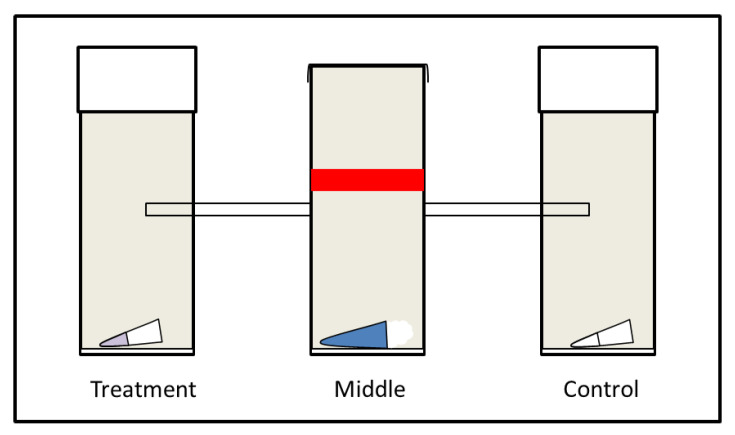
Experimental set-up of the two-choice trap-based bioassays that was used to test the attractiveness of identified *R. fruticosus volatiles*. The set-up consisted of three containers with a moist cotton pad placed on the bottom to provide sufficient humidity. At the start of each experiment, around 30 flies were released in the middle container, which contained ad libitum water and which was connected to the outer containers via tubes with a length of 6.5 cm. The outer container either contained an Eppendorf tube with a volatile compound identified from *R. fruticosus* (in an amount corresponding to that estimated to be present in 10 berries and dissolved in 100 µL of mineral oil) (treatment arm) or mineral oil solvent only (control arm). After 24 h, the overall attractiveness was assessed based on the number of flies present in each container.

To measure the attractiveness of each of the blackberry volatiles, a two-choice trap-based bioassay was used. Gravid female flies (4–10 days old) were used in the experiment. Males and female flies were anesthetised using CO_2_ and separated based on the presence or absence of the black wing spot, characteristic for *D. suzukii* males. The set-up consisted of three cylindrical polystyrene containers (Greiner Bio-one, container for plant tissue culture). The top of the middle container was closed off with a mesh, which allowed for sufficient air ventilation and prevented saturation inside the set-up. The outer containers were closed with a ceaprene stopper (Greiner Bio-one, Kremsmünster, Austria). The containers were connected with a 6.5 cm PTFE tube (6.35 mm ID × 7.94 mm OD, Cole-Parmer, Vernon Hills, IL, USA) (Figure 1). The tube connecting the containers was made to stick out 2 cm in the outer containers in order to create a trapping effect once the fruit fly made a choice [15]. A moist cotton pad was placed at the bottom of the three containers to prevent the flies from dehydrating. At the start of each experiment, around 30 flies were placed in the middle container. This container included a 1.5 mL Eppendorf tube (Eppendorf, Hamburg, Germany) with water and cotton wool to ensure a constant supply of water. The middle container also had a strip of red tape, as odors and color are known to be able to synergize [38] and preliminary experiments showed that this increased the response of the fruit flies. Subsequently, the flies were allowed to make a choice between the treatment and control arms in the bioassay over a total period of 24 h, while being placed under a 16:8 light:dark regime at 25 ± 1 °C. All containers were put in randomized orientation in open boxes with the sides covered to prevent any systematic biases in preference. 

When the experiment was finished, the containers were placed in the freezer and all flies were manually counted. From these counts, we then calculated attractivity, which was defined as the proportion of flies that chose the treatment vs. the control side, as well as a choice factor, which was defined as the proportion of flies that made a choice (i.e., choosing either the treatment or control container vs. those that made no choice, staying in the middle container). In between subsequent experiments, all containers and tubes were thoroughly cleaned with 70% ethanol and placed in an oven at 60 °C overnight to ensure that any remaining volatiles had evaporated. In principle, a volatile compound that showed a high and statistically significant attractive effect and also displayed a high choice factor was expected to potentially serve as a good lure. 

### 2.4. Behavioural Experiments with Rubus fruticosus Berries

To ensure that the frozen + cut condition of the blackberry in the sample did not decrease the attractivity compared to a fresh blackberry, an experimental two-way bioassay was conducted similarly as described above. These experiments were conducted 8 months before the experiments with the individual compounds, in a climate room (24 ± 1 °C) with a different lighting set-up. Four different conditions were tested: a whole berry, a cut berry, a frozen and cut berry and berry juice, always using the equivalent amount of one berry in the treatment container. Five replicate trials were carried out per condition.

### 2.5. Behavioural Experiments with Individual Compounds

The 33 commercially available volatile compounds were tested individually for attractiveness to *D. suzukii* using the experimental set-up described above. The tested compound was dissolved in 100 µL of mineral oil (Sigma, CAS: 8042-47-5) and placed in a 0.5 mL Eppendorf tube placed at the bottom of the treatment arm container, using a volume corresponding to that estimated to be present in 10 *R. fruticosus* berries. The mineral oil was used to allow each volatile to be released at a slow rate [27]. The control arm container was provided with a matching Eppendorf tube containing 100 µL of mineral oil solvent only. Six replicate experiments were carried out for each individual compound.

### 2.6. Statistical Analysis

To analyze the bioassay data two separate binomial generalized linear mixed models (GLMM) were used. In the first binomial GLMM, the proportion of flies that chose the treatment vs. the control side (=attractivity) were compared across all the tested compounds. In a second binomial GLMM, the proportion of flies that made a choice, i.e., choosing either the treatment or control container vs. making no choice and staying in the middle container, were compared (=choice factor). In both models, compound was included as a fixed factor. In the ‘no choice-choice’ model, the shelf on which the experiment was run was also included as a fixed blocking factor. Overdispersion was accounted for by incorporating each individual experiment as a random intercept. These binomial GLMMs were fitted using the *glmer* function in R’s *lme4* package. Tests against a 50:50 outcome (no preference) and pairwise FDR corrected posthoc comparisons among compounds were carried out using R’s *emmeans* package. 

## 3. Results

### 3.1. Identification and Quantification of R. fruticosus Volatiles

**Figure 2:**
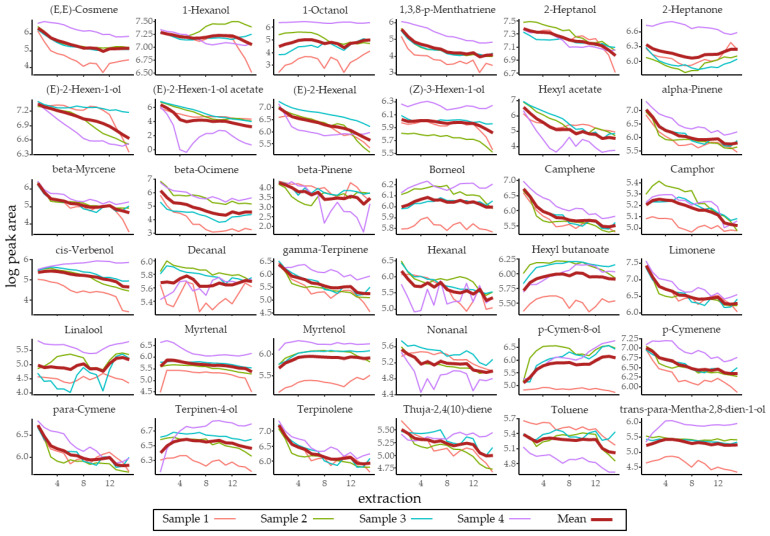
Measured peak areas over subsequent multiple headspace SPME extractions and GC-MS runs for all the identified *Rubus fruticosus* volatiles. The different samples are shown as a thin line. The thicker dark red line shows the geometric mean of the four samples, which was used to calculate the total peak area over the subsequent extractions, and which in combination with response factors measured from pure compounds allowed us to estimate the total amount (volume) of each volatile produced by a single *R. fruticosus* berry.

For the identification and quantification of the volatile compounds in *R. fruticosus*, four blackberries were analyzed using MHS-SPME GC-MS [30,31,32]. In total, 38 volatile compounds were identified in all samples, of which all except five that were not commercially available and another two that were highly volatile (ethanol and acetaldehyde) could be successfully quantified. The summed geometric mean peak area over the four replicate runs across 15 repeated extractions and GC-MS runs were used to estimate a total peak area, which in combination with the response factors measured from a liquid injection calibration run of pure synthetic compounds, allowed us to estimate the amount of each volatile produced by a single *R. fruticosus* berry (Table 1). For ca. half of all compounds, this curve showed a strong decline, as expected from the depletion of the volatiles still present in the sample following each subsequent extraction (Figure 2). For nine compounds (indicated as such with an asterisk in Table 1), no clear reduction of the measured peak area was observed over subsequent extractions. In this case, the estimated amount of those volatiles present in one berry were underestimated to some extent. To compensate for this, all bioassays were carried out with a volume corresponding to the estimated volume present in 10 blackberries (Table 1). For ethanol and acetaldehyde, where due to the high volatility, the exact amount produced by a single berry could not be estimated, a volume equal to that of the most abundant compound was used. The estimated volume of each volatile present in one berry ranged between 2 × 10^−6^ µL and 0.02 µL, with myrtenol being the most abundant volatile compound and camphor the least abundant one. 

### 3.2. The Effect of the Condition of R. fruticosus Berry on D. suzukii Attraction

**Figure 3:**
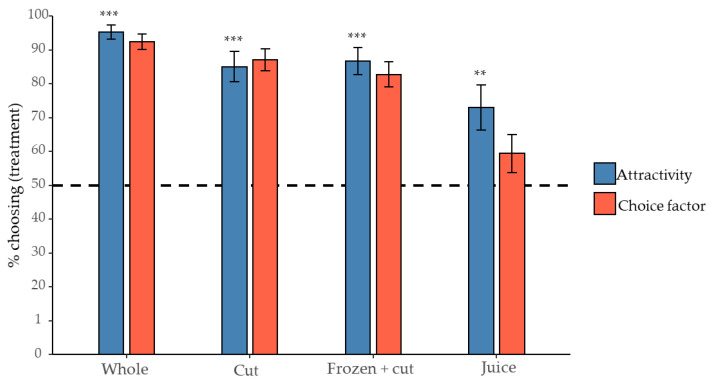
Results from two-choice preference tests of different blackberry conditions. Barplots in blue show the attractivity (=the proportion of flies that chose the treatment vs. the control side) of the blackberry condition. Attractivity was tested against a 50:50 distribution, shown as a horizontal dotted line (binomial GLMM, *FDR* corrected posthoc tests, mean ± SE, **: *p* < 0.01, ***: *p* < 0.001). Barplots in red depict the choice factor (=the proportion of flies that made a choice (choosing either the treatment or control container) vs. those that made no choice (remaining in the middle container)).

To validate if the cut and frozen condition of the berry as used in our GC-MS analyses did not decrease attractivity, blackberries were tested in four different conditions: whole, cut, frozen + cut or as juice. As expected, all the different conditions of *R. fruticosus* berry were significantly attractive to *D. suzukii* (*p* < 0.01). The whole berry was not significantly more attractive than a cut or a frozen and cut berry. In these conditions, an average of 85–95% of the flies preferred treatment over control. However, only 73% of the flies chose juice, which implied that the condition was significantly less attractive than the whole berry (*p* < 0.01) (Figure 3).

A second effect that was measured was the number of flies remaining in the central container (which did not make any choice) compared to the number of flies that chose for one of the external containers (treatment or control), referred to as the choice factor. Volatile compounds with a high and statistically significant attractive effect and a high choice factor were expected to represent a good basis for a potential lure. When juice was used, the choice factor was significantly lower compared to the other three conditions (*p* < 0.01). To a lesser extent, there was also a significant difference in the number of flies making a choice between whole berries and frozen + cut berries. On average more than 80% of the flies left the central container in the experiments with whole berry, cut berry and frozen and cut berry while in the samples with juice this was only 59% of the flies.

### 3.3. The Attractivity of Individual R. fruticosus Volatiles on D. suzukii

**Figure 4:**
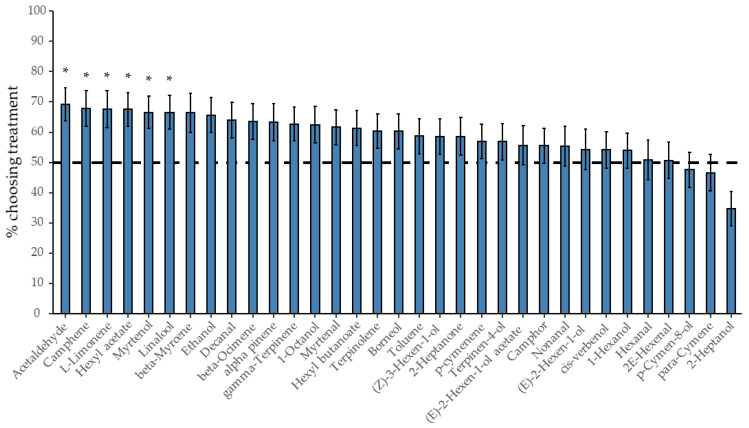
Results from two-choice preference tests of all the here identified and commercially available *Rubus fruticosus* volatiles. Column plot shows the attractivity (=the percentage of flies that chose the treatment vs. the control side) of each individual compound to *Drosophila. suzukii* females. Attractivity was tested against a 50:50 distribution, shown as a horizontal dotted line (binomial GLMM, *FDR* corrected, mean ± SE, *: *p* < 0.05).

All 33 commercially available volatile compounds were individually tested for their attractivity to female *D. suzukii* using a two-choice bioassay at volumes corresponding to the estimated volume present in ten *R. fruticosus* berries (Table 1). These assays identify six significantly attractive compounds: acetaldehyde, hexyl acetate, camphene, linalool, myrtenol and *L*-limonene (Figure 4). The mean proportion of flies choosing the treatment over the control ranged from 67% to 69% for these volatiles and there was no significant difference in the level of attractiveness among these six compounds. One compound, 2-heptanol, had a strong tendency towards a repellent effect, with 65% of the flies choosing the control side, although this repellent effect was strictly speaking marginally non-significant after *FDR* correction (*p* = 0.051). 

Acetaldehyde, hexyl acetate, linalool and myrtenol had the highest choice factor with an average between 71% to 78% of flies making a choice. The average choice factor of 54% and 58% for camphene and *L*-limonene was significantly lower than that of the other four attractive compounds. Except in comparison to acetaldehyde, *L*-limonene did not have a significantly lower choice factor (*p* = 0.07). Beta-myrcene was the compound where the lowest percentage of flies made a choice (choice factor = 42%), while it was the highest for *p*-cymen-8-ol (choice factor = 89%) (Figure S1). However, for *p*-cymen-8-ol, this high choice factor was not paired with high attractiveness.

## 4. Discussion

Our aim in this study was to identify the key compounds which mediate the attraction of *D. suzukii* to the berries of *R. fruticosus*–one of the preferred hosts of spotted wing drosophila [28,29]. Using MHS-SPME GC-MS we identified a total of 38 volatile compounds, half of them were already identified in a different *R. fruticosus* cultivar using similar detection methods [39]. 33 of the 38 identified compounds were commercially available and tested for *D. suzukii* attractiveness [30,31,32]. This resulted in six individual compounds that significantly attracted *D. suzukii*: acetaldehyde, hexyl acetate, camphene, linalool, myrtenol and *L*-limonene. Because acetaldehyde, hexyl acetate, linalool and myrtenol had a higher choice factor, i.e., causing a higher percentage of the *D. suzukii* flies to actually choose to move to either the control or treatment chamber, these compounds have the best potential of being a good lure. Earlier, hexyl acetate, limonene and linalool have also been shown to induce marked electroantennographic responses in *D. suzukii* [25,27,38,40,41]. As yet, no electroantennography, however, has been performed in any *Drosophila* spp. for any of the other three attractive components here identified. Nevertheless, the fact that we measure significant behavioral effects for those evidently implies that these compounds are sensed by the flies. In total, at least sixteen of the compounds identified here have been shown to be actively detected by *D. suzukii* [21,25,27,40,42,43], even though the detection of a single compound does not need to be associated with evoking a visible behavioral response [42]. Earlier, Keesey et al. [43] also showed that hexyl acetate is attractive to both *D. melanogaster* and *D. suzukii*. This reduces the utility of this compound for *D. suzukii* lures, as it might display poor selectivity. Another study documented a small but nonsignificant attractive effect of hexyl acetate on *D. suzukii* [41]. Dose-dependent behavioral effects and/or differences in statistical power to detect attractive effects with the setup used likely explain some of these discrepancies [42,44]. Lastly, hexyl acetate has also been shown to play an important role in the attraction of *Rhagoletis pomonella* [45]. Acetaldehyde is a known fruit fermentation product, originating as an intermediate in the conversion of ethanol to acetic acid [46]. Both acetaldehyde and limonene are known to be attractive to *Drosophila melanogaster* [47,48]. However, as yet, their attractive effect had not been demonstrated for *D. suzukii.* A four-component blend with limonene has been shown to have great potential for mass trapping the Mexican fruit fly (*Anastrepha ludens*) [49]. Linalool might be a good candidate to improve the attractiveness or selectivity of existing *D. suzukii* lures, already hinted at by Abraham et al., who identified this compound as one of the most abundant in raspberry [27]. In that study, raspberry was also shown to be the most attractive fruit to *D. suzukii* of all the tested fruits. Around the same time, linalool was shown to have a strong insecticidal effect on *D. suzukii* when used at a high concentration as a fumigant [50]. Linalool is also recognized by and has shown to be attractive to other Diptera such as Tephritidae and the housefly (*Musca domestica*) [51,52,53,54].

Because the experiments with the blackberries in different conditions (whole, frozen, frozen and cut or as juice) were carried out at a lower temperature and with a different lighting set-up, we cannot precisely compare these results with the experiments with individual compounds. However, it seems reasonable to assume that no individual compound could ever be as attractive to the fruit flies as the *R. fruticosus* berry itself. This could indicate that *D. suzukii* is attracted to the berries by a ratio-specific blend of volatiles rather than a single key compound, which is the case for many insects [16,55,56,57,58]. On the other hand, attractive effects of individual plant volatiles on host-specific insects (or a broad range of insects) are commonly observed as well [59,60,61]. Despite many efforts by us to combine volatiles in different concentrations in the naturally occurring ratios, we did not succeed in producing a blend that was more attractive than the single most attractive compound. A possible explanation could be that the structure of the fruit and associated matrix effects play an important role in the release dynamics of the compounds. This could also explain the observed difference in attractivity between a whole *R. fruticosus* berry and *R. fruticosus* juice. Nevertheless, the identification of attractive individual compounds can still be valuable as adding these to fermentation-based lures could potentially improve their attractivity or selectivity [27,62].

Quantification of the volatile compounds in this study was estimated based on the total peak area of each compound measured over fifteen repeated MHS-SPME extractions [30,31,32]. Because of the high number of extractions, we expected to observe a clear decline, which was not the case for all compounds. Although it proved to be effective for our experimental set-up to identify the attractive compounds, a more precise quantification method could also benefit future experiments (e.g., by using a smaller piece of berry, so that the SPME fiber would be less saturated [30,32]). However, this might then also result in some less abundant compounds not being picked up.

In conclusion, the results of this research can be a valuable starting point for the further development of improved *D. suzukii* lures with better attractiveness or species specificity. In the future, this could be accomplished either by creating a successful blend of the identified compounds or by addition of one or more of the attractive compounds identified here to already existing lures. In the future, further field trials to test such lures would be desirable.

## Figures and Tables

**Table 1:** List of all the volatile compounds identified and quantified via multiple headspace SPME GC-MS in *Rubus fruticosus*. The retention time (RT) and calculated retention index (RI) of each compound is shown. Summed peak area over fifteen repeated headspace extractions were calculated from the geometric mean sample average (*n* = 4 samples). Compounds that show no calculated volume were not commercially available or were too volatile to be quantified accurately (acetaldehyde and ethanol). Asterisks indicate compounds with no clear reduction in peak area over fifteen extractions.

Compound	RT	RI	Log(Peak Area)	Volume Present in 1 Berry (µL)	Volume Used in Bioassay (µL)
Acetaldehyde	0.93	408	-	-	0.2
Ethanol	1.02	446	-	-	0.2
Toluene	4.95	759	6.41	8.59 × 10^−6^	0.00009
Hexanal	5.90	793	6.85	7.94 × 10^−5^	0.0008
(E)-2-Hexenal	7.46	849	7.58	2.35 × 10^−4^	0.002
(Z)-3-Hexen-1-ol	7.65	856	7.13	7.93 × 10^−5^	0.0008
(E)-2-Hexen-1-ol	7.99	868	8.22	1.11 × 10^−3^	0.01
1-Hexanol	8.09	872	8.36	1.15 × 10^−3^	0.01
2-Heptanone *	8.55	888	7.32	1.21 × 10^−4^	0.001
2-Heptanol	8.93	902	8.39	1.87 × 10^−3^	0.02
beta-Pinene	9.50	919	4.9	-	-
alpha-Pinene	9.69	932	7.41	4.35 × 10^−5^	0.0004
Camphene	10.09	943	7.12	2.63 × 10^−5^	0.0003
Thuja-2,4(10)-diene	10.28	950	6.4	-	-
beta-Myrcene	11.38	987	6.52	1.11 × 10^−5^	0.0001
Hexyl acetate	12.05	1009	6.74	1.78 × 10^−5^	0.0002
(E)-2-Hexen-1-ol, acetate	12.13	1016	6.39	1.74 × 10^−5^	0.0002
para-Cymene	12.40	1025	7.26	3.60 × 10^−5^	0.0004
L-Limonene	12.53	1028	7.83	1.69 × 10^−4^	0.002
beta-Ocimene	13.19	1044	6.33	1.05 × 10^−5^	0.0001
gamma-Terpinene	13.56	1055	6.91	2.24 × 10^−5^	0.0002
1-Octanol *	14.13	1071	6.02	5.06 × 10^−6^	0.00005
Terpinolene	14.63	1084	7.55	7.35 × 10^−5^	0.0007
p-Cymenene	14.74	1088	7.75	7.55 × 10^−5^	0.0008
Linalool *	15.10	1101	6.12	6.29 × 10^−6^	0.00006
1,3,8-p-Menthatriene	15.61	1110	5.84	-	-
Nonanal	15.70	1115	6.35	6.72 × 10^−6^	0.00007
trans-para-Mentha-2,8-dien-1-ol *	16.03	1120	6.49	-	-
(E,E)-Cosmene	16.63	1134	6.68	-	-
Camphor	16.92	1141	6.32	2.42 × 10^−6^	0.00002
cis-Verbenol	17.10	1145	6.35	5.83 × 10^−5^	0.0006
Borneol *	17.98	1164	7.19	4.07 × 10^−5^	0.0004
Terpinen-4-ol	18.54	1176	7.69	1.84 × 10^−2^	0.2
p-Cymen-8-ol *	18.89	1183	7.05	5.84 × 10^−5^	0.0006
Hexyl butanoate *	18.99	1191	7.1	2.37 × 10^−3^	0.02
Myrtenal	19.36	1192	6.86	8.07 × 10^−6^	0.00008
Myrtenol *	19.53	1196	7.06	2.03 × 10^−2^	0.2
Decanal *	19.73	1205	6.83	1.57 × 10^−3^	0.02

## Data Availability

The data presented in this study are available on request from the corresponding author.

## Supplementary Materials

The following are available online at https://www.mdpi.com/article/10.3390/insects12050417/s1, Table S1: List of identified compounds that were commercially available and their suppliers, Figure S1: Column plot shows the choice factor (=the percentage of flies that made a choice (choosing either the treatment or control container) vs. those that made no choice (staying in the middle container)) of all the identified *Rubus fruticosus* volatiles using headspace SPME GC-MS that were commercially available. (binomial GLMM, *FDR* corrected, mean ± SE).
