# Supplementary material for: Identification of Blackberry (Rubus fruticosus) Volatiles as Drosophila suzukii Attractants
Source: Insects. 2021 May 6;12(5):417. doi: 10.3390/insects12050417 (PMC8148594; doi:10.3390/insects12050417)
Supplement: Supplementary file 1 [file insects-12-00417-s001.zip › insects-1183461-supplementary.pdf]

Supplementary Materials

Table S1. List of identified compounds that were commercially available and their supplier.

compound	CASnr.	company	purity
1-Hexanol	111-27-3	fischer sci	99%
1-Octanol	111-87-5	sigma-aldrich	99.7%
(E)-2-Hexenal	6728-26-3	sigma-aldrich	98%
2-Heptanol	543-49-7	acros organics bipp	99%
2-Heptanone	110-43-0	vwr	99%
(E)-2-Hexen-1-ol	928-95-0	sigma-aldrich	96%
(E)-2-Hexen-1-ol, acetate	2497-18-9	sigma-aldrich	98%
(Z)-3-Hexen-1-ol	928-96-1	sigma-aldrich	98%
Acetaldehyde	75-07-0	sigma-aldrich	99%
Hexyl acetate	142-92-7	sigma-aldrich	99%
alpha-Pinene	80-56-8	acros organics bipp	97%
beta-Myrcene	123-35-3	sigma-aldrich	90%
beta-Ocimene	527-84-5	sigma-aldrich	98%
Borneol	507-70-0	Fufjifilm	70%
Camphene	79-92-5	sigma-aldrich	95%
Camphor	76-22-2	sigma-aldrich	96%
cis-Verbenol	1845-30-3	sigma-aldrich	95%
Decanal	112-31-2	sigma-aldrich	98%
Ethanol	64-17-5	Fischer chemical	99%
gamma-Terpinene	99-85-4	sigma-aldrich	97%
Hexanal	66-25-1	sigma-aldrich	98%
Hexyl butanoate	2639-63-6	sigma-aldrich	98%
Linalool	78-70-6	sigma-aldrich	97%
L-Limonene	5989-54-8	sigma-aldrich	96%
Myrtenal	18486-69-6	sigma-aldrich	98%
Myrtenol	515-00-4	sigma-aldrich	95%
Nonanal	124-19-6	sigma-aldrich	97%
para-Cymene	99-87-6	sigma-aldrich	99%
p-Cymen-8-ol	1197-01-9	sigma-aldrich	95%
p-Cymenene	1195-32-0	sigma-aldrich	98%
Terpinen-4-ol	562-74-3	sigma-aldrich	95%
Terpinolene	586-62-9	TCI europe bipp	90%
Toluene	108-88-3	FS, part of TFS bipp	99.85%

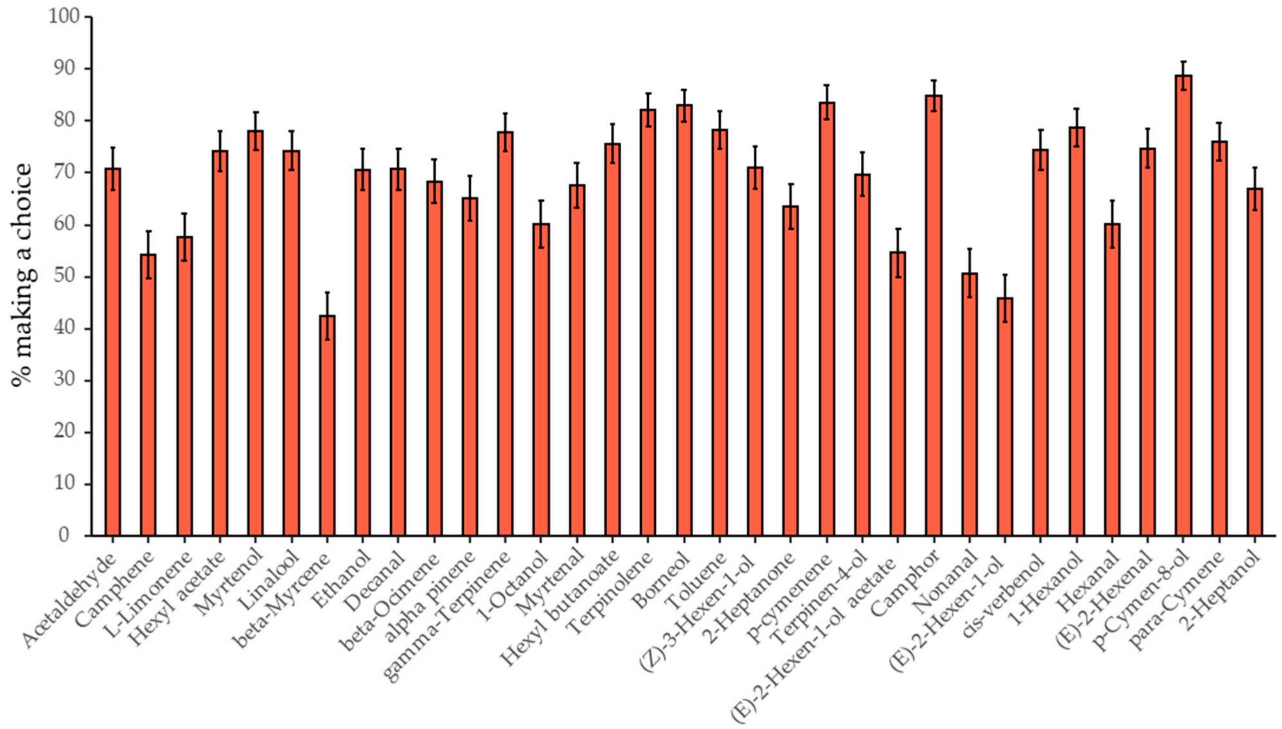

Figure S1. Barplots depicting the choice factor (= the proportion of flies that made a choice (choosing either the treatment or control container) vs. those that made no choice (staying in the middle container)) of all the identified *R. fruticosus* volatiles using headspace SPME GC-MS that were commercially available. (binomial GLMM, *FDR* corrected, mean ± SE).
